# Surgical Treatment of Tubercular Peritonitis1Read at the thirtieth annual meeting of the Medical Association of Central New York, at Buffalo. October 19, 1897.

**Published:** 1898-01

**Authors:** J. P. Creveling

**Affiliations:** Auburn, N. Y.


### SURGICAL TREATMENT OF TUBERCULAR PERITONITIS.[1]

By J. P. CREVELING, M. D., Auburn, N. Y.

IT IS comparatively recent that surgical means have become a recognised treatment in tuberculosis of the peritoneum, and the general scope, as well as the ultimate extent to which such measures may be resorted to with benefit, still remain problems for further demonstration. The brilliant results we see in some instances are somewhat clouded by the utter failure in others, but any means that presents even a fair prospect of giving more or less permanent beneficial results in a disease so uniformly destructive as tuberculosis, wherever situated, must be regarded with favor.

In a paper of this character, it is not necessary to enter into the classification of the various forms of peritonitis, as it is of but little significance to the surgeon operating whether it be given an anatomic, pathologic, etiologic or an anatomo-bacteriologic classification, so long as he recognises the conditions before him and proceeds with care and prudence to overcome the malady calling for operative intervention. I would not be understood as disclaiming the value of scientific classification of this, as well as all other diseases, but the various forms of inflammation occurring within the peritoneum or its cavity are in many instances extremely difficult of differentiation before the abdomen is opened, and if the surgeon has made good his diagnosis as to the tubercular nature of the case, he has quite well discharged his duty as far as diagnosticating the disease is concerned.

It is true, of course, that when the disease involves the mesentery, the details of the required manipulations may be somewhat

1. Read at the thirtieth annual meeting of the Medical Association of Central New York, at Buffalo, October 19, 1897.

different from those when it is located within the pelvic cavity or has invaded the peritoneum lining the walls of the cavity. Many of these cases, when they are first presented to the surgeon, are more or less in an advanced stage, so that the morbid process is not confined to any one part, but the whole abdominal contents have become involved in varying degrees. All the viscera may be firmly agglutinated and held together in one mass and the whole peritoneum intensely inflammed. In such cases, it is impossible to locate the point of origin.

The most important question to determine before operation is whether the deposit of tubercle is confined to the abdominal viscera or whether the peritoneal involvement is only a part of a general tuberculosis. If this be the case, I believe, as a rule, it is inadvisable to operate, but there are exceptions to this.

In tubercular joint disease associated with a similar peritoneal process, a double operation, removing the diseased bone and performing laparatomy at the same time, or the two operations separately, will in many instances give a long period of relief, if not entirely eradicate the disease. Such has been my experience in a limited number of cases.

In one of my cases, where there was slight pulmonary involvement, the result was very satisfactory. All the abdominal indications ceased and the pulmonary has progressed but slowly, thus giving the patient a much longer period of life and comfort than would otherwise have been possible.

Of course, the result in such cases is always a problem, and the advisability of operating should be determined by a careful consideration of all the surrounding circumstances; but it should not be forgotten that intervention is sometimes justifiable merely as a palliative measure, and that the severity of the operation does not preclude its adoption even in cases admitting of some doubt.

It is in the purely local form of the disease, however, that we may look for the most satisfactory results. Here, we should not be discouraged because the disease is extensive. If the general condition of the patient will admit of the operation, I believe it the proper thing to do, as it holds out the best prospects of relief, and it is this very class of cases that is liable to yield the most brilliant results. But they, too, require patience and diligence, as in most instances there will be found extensive adhesions, which should be sought and divided, if possible. The more thoroughly this is accomplished, the better will be the chance for a complete cure, but care should be exercised lest the intestinal wall be injured. These adhesions may be so generally diffused and of sufficient strength to immobilise the bowels, which in turn are much distended by gas and weakened by disease, and, therefore, much more liable to rupture than under ordinary circumstances. Hemorrhage may be quite profuse if the adhesions are thick and well organised and should be securely controlled before closing the incision.

The advisability of removing the bowels from the abdominal cavity is questionable. While it makes a more thorough exposure, it increases the shock and risk of rupture, as a large mass of bowels fully distended by gas and suffering from paresis are not always as easily returned to their normal cavity as removed, but all tubercular foci should be searched out and, if not removed, should be incised or curetted, as manual disturbance seems in some way to enhance the chances of a satisfactory result.

In cases of general fibro-plastic peritonitis, numerous deposits of tubercle may be found in the organised exudate as well as in the abdominal viscera. In a case I operated on a few weeks since, this material was studded with tubercular nodules. This was a case, however, of secondary development, as one arm had been amputated some months before for a similar disease at the elbow. In this, the exudate was greater than I had seen before. The bowels were all adhered to each other and all firmly bound to the posterior peritoneal wall. Over the whole mass was an apron-like covering of organised plastic material, completely enclosing all the bowels and dividing the cavity into an upper and pelvic portion. In the pelvic peritoneal space was confined 168 ounces of serum. There was considerable oozing of blood during the separation of the intestinal coils, which was rather tedious, because of the strength of the adhesions. Some required the aid of the knife. The cecum could not be liberated. Although the operation was a long one, the shock was not unusually pronounced. The abdomen was not irrigated, but carefully dried, a drainage-tube inserted and the wound closed, which healed by primary union. The tube was removed on the third day, as there had been no drainage for twenty-four hours previous. This was an unfavorable case for operative measures, as the lesions were so extensive they could not all be reached and the disease will undoubtedly soon manifest itself again. It serves to illustrate the tolerance of the peritoneal cavity to manual manipulation and at the same time has prolonged and rendered life more comfortable.

In a laparatomy I performed a year or so ago for another purpose, a large section of the omentum was discovered to be tuberculous. It was ligated and removed and as yet there has been no apparent return of the disease.

When such radical means can be safely adopted, the result of the operation is much better guaranteed than by less thorough methods, as for instance simple air exposure and the like.

The question as to how or by what means simple laparatomy proves beneficial is still in doubt. Mere exposure to the air in many instances effects a complete cure. In fact, it is claimed, and statistics seem to support the claim, that about 70 per cent. of all cases are cured by this means. (Roesch has collected 358 cases operated upon, of which number 250 were cured.) Morris, of New York, I believe, claims the result is due to putrefactive changes caused by bacteria from the air; and I understand he has taken the fluid from the abdominal cavity and after exposure to the air obtained a toxalbumin which is very fatal to the tubercle bacillus. Thomson, of Edinburgh, believes the initial incision is the essential factor in bringing about the curative result. This opinion is shared by others. Nannotti and Baciocche, after some experiments, attribute it to the mechanical influence stimulating the reparative changes in the peritoneum. Stchegoleff believes recovery is reached by the inflammatory process, that the phagocytes play the important rôle. Jordon concludes the real cause is undetermined. It is held by some that the evacuation of the fluid is a potent factor, but, then, the disease occurs occasionally without fluid.

Whatever the curative influence may be, what experience I have had has led me to believe that in fibro-plastic cases the adhesions should be carefully but thoroughly broken up, if possible; that mere exposure to air is insufficient, or at best it does not insure the favorable chances that a more complete operation does; and, on the other hand, the danger encountered is not as great as in a prolonged exposure of the intestines to the air. It also diminishes the liability of contraction of the fibrous deposit, and upon the whole is far more surgical.

In simple cases—that is, in cases not associated with the foregoing complications,—there seems to be but little doubt that mere air exposure is adequate to effect the desired result.

Lately, the introduction of sterilised air into the abdominal cavity has been tried and some rather encouraging results reported, especially in children. Now, sterilised air is not supposed to contain many putrefactive bacteria, and neither should the mechanical manipulations in introducing a trocar through the abdominal walls create a great deal of inflammatory reaction. The question, therefore, really still remains open for further demonstration.

22 South Street.

DISCUSSION.

Dr. Jacobson, of Syracuse.—My own experience with the surgical treatment of tubercular peritonitis covers a period of something like eight years. A case operated upon at that time is still alive and in perfect health. There was no question at all about the diagnosis after the abdomen was opened, but there was some doubt whether it was a tubercular peritonitis before the operation. It was one of the forms where the adhesions were so marked that the growth seemed a great deal like a cystic tumor which had fixed adhesions. In that case nothing was done but to open into this accumulation, thoroughly carry off all of the fluid, mop it out, dry and close it up. The woman had had high fever prior to the operation and for a week afterward the fever continued. Since that time she has had no further evidence of tubercular trouble, although every autumn she is put upon cod liver oil and continues it until spring.

I have had other cases recover, none, however, showing the period of immunity that this case presents. On the other hand, I have had a number of cases which have gone on to death, and in some instances I have rather been of the opinion that the operation has hastened death. I cannot agree with the suggestion of the paper quoted from Dr. Morris, that opening into a tubercular cavity favors its cure, because of the introduction of a mixed infection. Now, we know that is absolutely contrary to all surgical teachings. We know that tubercular abscesses about joints do splendidly sometimes by a let-alone or conservative treatment, until an incision is made. Immediately afterward we frequently have to deal with one of the most virulent forms of infection. Men who have studied this subject, especially Watson Janes and Sims Woodhead, in England, are satisfied that mixed infections in tubercular disease increase the trouble. Therefore, it seems to me that a mere statement of this kind, without further evidence that it is true, is insufficient to demand from us its acceptance as the theory of the cure of these cases. We do not know wherein the cases recover. And there is the other element—we are not always sure that we are dealing with a tubercular disease.

The mere presence of tumors, or tumorous masses, or tuberculous masses, which represent in their gross appearance tuberculosis, or the larger form of tubercular nodules, is not evidence enough that they are positively due to tubercle bacilli. There must be some further evidence than this. Therefore, it may be possible that a number of these cases get well simply as an operation, *per se*, as many other forms of tumorous troubles in the abdomen recover. We cannot say yet what has cured some of these cases. And I presume others have had the same experience as I have—namely, that they have predicted the early death of cases which to all external appearances were cases of tubercular peritonitis and to their happy surprise they have found them get well without operation. It has happened to me three times that where I was satisfied I had to deal with tubercular peritonitis, operations were refused and the patients got well. Now, I cannot help but believe that there was a faulty diagnosis there, and yet there was a group of symptoms present in each case that seemed to justify such a conclusion.

Dr. CHEESMAN, of Auburn.—There is perhaps one point we might take issue on—namely, that the local forms of tubercular diseases are more amenable to treatment than the general forms. I think the statistics that the author has quoted refer in the great majority of instances to cases of general abdominal infection, to general abdominal tuberculosis, and certainly, personally, it would seem to me an easier matter to treat such a case by a simple laparatomy and irrigations, perhaps, than it is to handle a tuberculous mass localised in the abdomen. My own experience with such cases is that they are very difficult to deal with.

I have a case on hand now with a localised tubercular trouble behind the uterus, in which there is general infection. Laparatomy seems to have done away with the general tuberculosis of the abdomen so far as can be judged by the present condition of the patient, but the localised trouble is a very serious problem just now, as there is a discharge from a mixed infection from this collection behind the uterus. I don't think we know precisely why we do get recovery after laparatomy in general peritonitis.

All sorts of theories have been offered to account for it, —ventilation and the washing out and the handling and the oxygenation of the peritoneum, and various other schemes have been suggested, —but I do not think anything has stood the test of investigation as yet. At any rate, clinically we do know that local forms of general peritonitis do very well when they are simply washed out.

Dr. LICHTY, of Clifton Springs.—I have had no practical experience with the subject of tubercular peritonitis, but theoretically it is a question of great interest to me, especially after having read Dr. Victor C. Vaughn's article.

I think the statistics prove conclusively that tubercular peritonitis is cured after abdominal section. In patients who have been operated upon for this disease and afterward the second time for other reasons, tubercles were not found which were observed the first time ; and also in autopsies this same observation has been proved. Dr. Vaughn's theory is that it was due to leucocytosis, but that, of course, is something which needs to be proven. I wish further to state that he in that article also said that in cases following sarcoma or sarcomatosis of the peritoneal cavity, later found that the sarcoma had disappeared. In a patient of my own, about a year ago, upon whom the late Dr. Baker of Auburn operated, there was a sarcomatosis. This was proven by microscopical examination afterward. The patient had passed from my charge and was sent home to die, but I am hearing that the patient is getting well.

Dr. MANN, of Buffalo.—I have had considerable experience in tubercular peritonitis and have seen a good many cases get well after merely opening the abdomen and drying it out thoroughly. I have seen other cases, however, which did not get well, and I think there is a line of distinction, some points at least, where we can distinguish between those that are going to get well and those which will not get well. Where the abdominal viscera are not too firmly glued together so as to admit of their being separated and will come down and fill up the space which is made by taking out the water, and the entire abdomen is restored to a healthy anatomical condition, one might say, I think in such a case we have a very good chance of recovery ; but I have seen two cases where the viscera were so firmly glued together that it was absolutely impossible to separate them. When the water was taken out, therefore, there was nothing to fill up the cavity. The pelvis and the lower part of the abdomen was simply a big hole in which watery fluid and a little blood accumulated, decomposition took place and the patient died of sepsis.

There is another class of cases which is even more likely to be fatal than these and they are cases where there is already a mixed infection before the abdomen is opened. I have seen three such that I can recall. One was a trained nurse, one is a patient in the general hospital at present, where an abscess formed between the folds on the intestines, where there was tubercular peritonitis everywhere, where there was no ascites, but a distinct abscess formation. In the case of the trained nurse there was an abscess between the folds of the small intestine. This was opened and drained, but she died. Very possibly there were other abscesses deeper down of a similar nature which I couldn't get at. Then there is one in the general hospital now. She may possibly get well ; she is improving a little. She is taking nuclein by the way and she has an abscess just on top of the uterus, independent of the tubes and ovaries, but between the bladder and the uterus and the abdominal wall and intestines. There is general tuberculosis beside, but no ascites. These are the cases which are very difficult to manage, as the author says, but where there is a mixed infection and a more localised trouble they are very difficult cases and cases which are not so apt to get well. I simply speak of them from the clinical aspect and not theoretically—that those where the abdomen is opened and a large quantity of fluid removed, where the intestines can be brought down to fill the space, have a very good chance to get well. I have seen a dozen of them get well after operation; but the other cases are not so apt to recover.

Dr. Elsner, of Syracuse.—I had an experience about a year ago at our hospital in Syracuse, which seems to throw still further doubt upon the method of cure in these cases, and seems to prove almost conclusively that the mere admission of the air and added infection is not really the agent of curing many of these forms of tubercular peritonitis. We had a young Italian who came in with an acute, far-reaching miliary tuberculosis; he had at the same time tubercular peritonitis, characteristic body feel in the upper half; in the lower half he had an abundance of fluid; he had besides this a tuberculous pleurisy. He was operated upon; the fluid from the abdomen was allowed to escape; the intestines in the upper half were found adherent; nothing further was done. We thought at that time of emptying out both pleural cavities of the fluid therein contained, but concluded to wait and see what the operation on the abdomen would do for the case. To make a long story short, the pleurisy disappeared with all abdominal symptoms and he is today perfectly well. This case ran its course in acute symptoms, commenced between three or four weeks before the operation. It is very strange, however, that this pleurisy and all evidence of pulmonary and pleural tuberculosis disappeared after the operation.

In this case there could have been no doubt of the diagnosis. In many cases I must fully agree with Dr. Jacobson in regard to the diagnosis. Even after we have opened the abdominal cavity, I think Welch, of Baltimore, and Richardson, of Boston, have shown conclusively that oftentimes we find these nodules in the peritoneum, and they are not tubercle nodules, but simply fibrous growth as the result of a long-continued latent peritonitis. Still further, the statistics show, and the very exhaustive paper which appeared a number of years ago, written by Osler, in the Hopkins hospital reports shows, that those cases are favorable where there is a good deal of fluid, and that is the general view taken today by clinicians; that those cases where we have far-reaching agglutinations which bind them and which cannot be moved are the unfavorable cases, whereas with plenty of fluid you can be pretty sure that you are going to have a better result.

Dr. Creveling (closing the discussion).—I was about to say as I closed my paper, that I do not agree with Dr. Morris with reference to his theory of the mixed infection or the putrefactive bacteria infection. Of course, it is very much of a question at the present time what is the cause of the cure. Now, if sterilised air cures the trouble, why then surely it is not due to a putrefactive bacteria entering the abdominal cavity, or at least should not be so, and neither should it be due to the mechanical effect of operations, because there should be but very little reaction following a trocar introduced into the abdomen. Now, as a reference to diagnosis: we often see little hard nodules that to the naked eye seem very much like tubercles; they look and feel like them and the only way to discriminate is to put them under the microscope and see whether they contain the characteristic formation of tubercle or the bacillus. Regarding cases of spontaneous cure that we see reported, I have never seen one and I have but little confidence in their occurrence.

A word as to the adhesions: I agree with Dr. Mann that wherever adhesions can be broken up, that it should be done. Of course, there are cases where they are so strong, where the bowel has become so weakened with the disease that it cannot be done and it would be unwise to rupture a bowel to break up an adhesion, but in all those instances where it can be done I believe it is the proper thing to do. This is the point I wish to make in the paper and I have post-mortem observations supporting the same.

